# Hyperbaric Oxygen Therapy as a Sole Agent Is Not Immunosuppressant in a Highly Immunogenic Mouse Model

**DOI:** 10.1155/2011/579268

**Published:** 2010-08-03

**Authors:** Adam Gassas, Weixian Min, A. Wayne Evans, Susan Carter, George K. Sándor, Eyal Grunebaum

**Affiliations:** ^1^Division of Haematology/Oncology/BMT, The Hospital for Sick Children, University of Toronto, 555 University Avenue, Toronto, ON, Canada M5G 1X8; ^2^Division of Immunology and Allergy, The Hospital for Sick Children, University of Toronto, ON, Canada M5G 1X8; ^3^Hyperbaric Medicine Unit, University Health Network, Toronto General Hospital, Faculty of Medicine, University of Toronto, ON, Canada M5G 2C4; ^4^Animal Facilities, Faculty of Dentistry, University of Toronto, Toronto, ON, Canada M5G 1G6; ^5^Oral and Maxillofacial Surgery, University of Toronto, Toronto, ON, Canada M5G 1G6; ^6^Tissue Engineering, Regea Institute for Regenerative Medicine, University of Tampere, 33520 Tampere, Finland

## Abstract

*Background*. Hyperbaric oxygen (HBO) therapy, which is used for many conditions, may also have immunosuppressive effects and could be used for prevention or treatment of graft-versus-host disease (GvHD). If HBO is immunosuppressant, then we hypothesize that HBO therapy will delay the T-cell mediated skin graft rejection. *Methods*. C57/BL6 black-coated (H2B) mice received skin graft from CBA (H2D) white-coated mice. Mice were treated with either 19 session of 240 kpa oxygen or 29 session of 300 kpa oxygen, for 90 minutes. Mice were housed either 4 per cage or separately, to prevent friction and mechanical factors that may affect graft survival. Skin grafts were assessed daily. *Results*. There was no difference in length of graft survival between mice that received either regimens of HBO therapy and mice that did not receive HBO therapy. *Conclusions*. HBO therapy, as a sole agent, did not delay skin graft rejection in a highly immunogenic mouse model.

## 1. Introduction

Exposure to HBO therapy, at least in rodents, appears to be immunosuppressant and leads to an anti-inflammatory effect. Several mechanisms have been proposed; inhibition of interferon-*γ* [1], interleukin-1 *β*, and tumor necrosis factor *α* release [2], a temporary drop in the CD4 : CD8 lymphocyte ratio [3], and a decrease of lymphocyte proliferation [4]. As a result, HBO exposure attenuates the immune system, increases the susceptibility to respiratory infections [5, 6], and delays allograft rejection in mice [7–9]. On the contrary, other reports suggest no immunosuppressant effect of HBO or even immunostimulant effect in particular neutrophiles activation [10, 11]. At present, there is a wide spectrum of indications for HBO therapy. According to the Undersea and Hyperbaric Medical Society, HBO therapy is indicated for carbon monoxide poisoning and gas embolism [12], clostridial myonecrosis [13], compartment syndrome and other traumatic ischemia with nonhealing wounds [14], necrotizing soft tissue infections, sepsis, and others [15–18]. Given the increasing availability of HBO therapy in recent years and the rarity of serious side effects, HBO therapy as an immunosuppressant is an attractive option. Perhaps, HBO therapy could be added to prevent or to treat GvHD in allogeneic hematopoietic stem cell transplantation (HSCT). Therefore, our aim was to test the effect of HBO therapy on the immune system by measuring the integrity and the duration of skin allogeneic graft in mice. Graft rejection is a T-cell mediated phenomenon, and if HBO therapy is immunosuppressant, then exposure to HBO therapy could potentially delay graft rejection.

## 2. Materials and Methods

Six-eight week-old black-coated C57/BL6 (H2B) mice, purchased from Jackson Laboratory, received skin grafts from CBA (H2D) white-coated mice donors. Mice were randomly assigned to either receive HBO treatment or not (control). All experiments were approved by the animal facilities Research Ethics Board, University of Toronto.

Skin grafting was performed accordingly, as previously described [19]. Briefly, mice received a 0.5 × 0.5 cm full thickness skin tail graft under general anesthesia. One donor mouse produced grafts for 4 recipients. Grafts were secured with Vaseline gauze and a bandage and sutured with 5-6 sutures of 6–0 prolene in at least the four corners. Two regimens of HBO therapy were used. In the first regimen, HBO treated mice received 2 HBO sessions the day prior to the skin grafting, one HBO session on the day of the skin grafting followed by twice daily sessions 5 days every week for total of 19 sessions. Each HBO session consisted of 90 minutes exposure to 100% oxygen at 2.4 atmospheres pressure. In this group, 4 mice were housed in one cage at all times. In the second HBO therapy regimen, oxygen pressure was increased to 300 kpa and mice received a total of 29 sessions, including 10 sessions (2/day for 5 days) in the week prior to skin grafting, one session during the day of the skin graft followed by 2 sessions per day, 5 days per week for 2 weeks. In the second HBO therapy group and in the additional control group, each mouse was housed in a separate cage to prevent potential overcrowding and mechanical factors such as friction that may affect skin graft survival. The HBO chamber allows only 2 cages at each session, therefore in both regimens, 4 mice shared one cage during HBO therapy. Skin graft survival was assessed daily by 2 separate examiners. Kaplan-Meier graphs were constructed for graft survival and log-rank comparisons of the groups were calculated. *P* < .05 was considered statistically significant.

## 3. Results

Transplanted mice received 2 regimens of HBO therapy. In the first regimen, among the 8 mice that received 19 courses of HBO, skin graft was lost after 7.1 ± 1.4 days (range: 5–9 days), compared to graft loss after 8.4 ± 1.7 days (range: 6–11 days) in the mice that did not receive HBO therapy (*P* = .12). In the second regimen, higher oxygen pressure (300 versus 240 kpa), increased number of HBO therapy sessions (29 versus 19 sessions), and reduced mice crowding (1 mouse versus 4 mice per cage) were employed with the addition of 8 mice in each of the HBO treatment group and control group. In the HBO therapy group, one mouse suffered a seizure-like episode after 3 HBO sessions and was euthanized. In the remaining HBO-treated mice, skin graft was rejected after 6.3 ± 2.4 days (range: 5–9 days) compared to 7.4 ± 1.6 days (range: 4–11 days) in the control group (*P* = .32).

## 4. Discussion

To study the potential use of HBO therapy in modifying graft rejection, we employed a well-recognized skin graft animal model. In this study we found that HBO therapy as a sole agent was not immunosuppressant enough to cause delay in allo-skin graft rejection. Our experiment was repeated twice to allow further testing of individual housing of mice in order to prevent mechanical factors affecting the graft survival. Our attempts to increase oxygen pressure to 300 kpa did not prolong graft survival, further suggesting that HBO had no significant anti-inflammatory effect in our model. Moreover, one of the mice treated with the higher oxygen pressure experienced a seizure episode. 

Several investigators have reported the likely immunosuppressant effect of HBO therapy. In 1979, Jacobs et al. reported that a mouse ear composite allograft rejection was significantly delayed by exposure to HBO therapy [20]. Since then, many other investigators published similar reports [21, 22]. However, after more than thirty years from the original report, HBO therapy is not utilized for its immunosuppressant effect as a sole or an adjuvant agent for human clinical use. That has raised some concerns among clinicians and researchers alike regarding translating these animal studies into human clinical use. Hence, our experiment was designed to build up an animal model with 2 different HBO atmospheric pressures, short or prolong exposure to HBO along with the elimination of mechanical factors such as friction by housing one mouse per cage for the study and control mice, and if the animal model is successful, then to try HBO therapy as an adjuvant agent for graft-versus-host disease prophylaxis in very high risk patients receiving unrelated hematopoietic stem cell transplantation. However, there was no evidence of immunosuppression in our mouse model. We hypothesize that HBO therapy may be immunosuppressant by the different mechanisms described by others. However, the immune system is so complex and includes many other factors and cytokine signaling that may play a counter effect. For example, HBO may reduce CD4 : CD8 lymphocyte ratio [3] and decrease lymphocyte proliferation [4] on one hand but increases other cytokines or signaling effects that may activate other parts of the immune system with no resultant significant immunosuppression on the other hand. 

The beneficial effect of HBO therapy in autologous grafting or flaps in animals is remarkable, and it is clear that HBO increases the tolerance of tissue to ischemia, increases the viability of tissues, and helps in the early neovascularisation [23–25]. The clinical applicability of HBO for composite autografts has been reported by many other surgeons and, most impressive of all, was the successful reimplantation of a near-total amputation of the nose followed by HBO therapy given twice daily in a 2-year-old girl [26], as was the reimplantation of the tip of the nose after a dog bite in a 5-year-old boy [27]. This potential which increases ischemia tolerance and improves graft survival from the autograft studies by HBO therapy was not noticeable in our mouse skin allograft. Our hypothesis is that in our skin allograft mouse model, the immune-mediated rejection and the resultant necrosis have masked the subtle potential benefits of increasing tolerance to ischemia.

In conclusion, our data did not support the hypothesis of HBO therapy as an effective sole immunosuppressant agent. Perhaps, HBO therapy may be immunosuppressant if combined with other immunosuppressant agents rather than a sole agent. Further research testing different combinations of immunosuppressant agents plus HBO is warranted.
